# Should we use diastolic function parameters to determine preload responsiveness in cardiac surgery? A pilot study

**DOI:** 10.1186/s44158-021-00014-7

**Published:** 2021-10-30

**Authors:** Athanase Courbe, Clotilde Perrault-Hébert, Iolanda Ion, Georges Desjardins, Annik Fortier, André Denault, Alain Deschamps, Pierre Couture

**Affiliations:** 1 Department of Anesthesiology, Montreal Heart Institute, Université de Montréal, 5000 Belanger Street, Montreal, Quebec H1T 1C8 Canada; 2 Department of Montreal Health Innovations Coordinating Center, Montreal Heart Institute, Université de Montréal, Montreal, Quebec Canada

**Keywords:** Cardiac surgery, Fluid responsiveness, Left ventricular diastolic function

## Abstract

**Background:**

Left ventricular (LV) diastolic function (DF) may play an important role in predicting fluid responsiveness. However, few studies assessed the role of diastolic function in predicting fluid responsiveness. The aim of this pilot study was to assess whether parameters of right and left diastolic function assessed with transesophageal echocardiography, including the mitral E/e′ ratio, is associated with fluid responsiveness among patients undergoing elective bypass graft surgery. We also sought to compare other methods of fluid responsiveness assessment, including echocardiographic and hemodynamic parameters, pulse pressure variation, and stroke volume variation (SVV) (arterial pulse contour analysis, Flotrac/Vigileo system).

**Results:**

We prospectively studied seventy patients undergoing coronary artery bypass grafting (CABG) monitored with a radial arterial catheter, transesophageal echocardiography (TEE), and a pulmonary artery catheter (for cardiac output measurements), before and after the administration of 500 mL of crystalloid over 10 min after the anesthetic induction. Thirteen patients were excluded (total of 57 patients). Fluid responsiveness was defined as an increase in cardiac index of ≥ 15%. There were 21 responders (36.8%) and 36 non-responders (63.2%). No difference in baseline pulsed wave Doppler echocardiographic measurements of any components of the mitral, tricuspid, and pulmonary and hepatic venous flows were found between responders and non-responders. There was no difference in MV tissue Doppler measurements between responders and non-responders, including E/e′ ratio (8.7 ± 4.1 vs. 8.5 ± 2.8 in responders vs. non-responders, *P* = 0.85). SVV was the only independent variable to predict an increase in cardiac index by multivariate analysis (*P* = 0.0208, OR = 1.196, 95% CI (1.028-1.393)).

**Conclusions:**

In this pilot study, we found that no parameters of right and left ventricular diastolic function were associated with fluid responsiveness in patients undergoing CABG. SVV was the most useful parameter to predict fluid responsiveness.

**Trial registration:**

ClinicalTrials.gov, NCT 02714244. Registered 21 March 2016—retrospectively registered.

## Background

Fluid responsiveness after volume expansion is better predicted by dynamic parameters compared to static parameters [1–3]. Among dynamic parameters, pulse pressure variation (PPV) and stroke volume variation (SVV) during mechanical ventilation as calculated by the FloTrac system algorithm has comparable sensitivity and specificity [4–7] to predict an increase in cardiac output after fluid administration.

The Frank-Starling curve illustrates fluid responsiveness, which depends on preload but also on systolic and diastolic ventricular function, venous compliance, and ventricular compliance [3, 8]. However, few studies assessed the role of diastolic function in predicting fluid responsiveness [9, 10]. Using TEE, the E velocity to mitral annular early diastolic velocity (e′) ratio (E/e′) is the measure of diastolic function that is best correlated with left ventricular filling pressure [11–13]. The E/e′ ratio is more representative of filling pressure, and potentially preload, because the e′ velocity corrects the effect of ventricular relaxation on E velocity [8]. So far, the association of the E/e′ ratio with fluid responsiveness has never been studied in cardiac surgical patients. In addition, no study evaluated multiple parameters of right and left diastolic function in their ability to predict fluid responsiveness. Our hypothesis is that parameters of right and left diastolic function measured with TEE, including the mitral E/e′ ratio, reflects preload reserve and is associated with preload responsiveness.

The aim of this pilot study was to assess whether parameters of right and left diastolic function, including the mitral E/e′ ratio, is associated with fluid responsiveness among patients undergoing elective bypass graft surgery. We also sought to compare other methods of fluid responsiveness assessment, including echocardiographic and hemodynamic parameters, PPV, and stroke volume variation (SVV).

## Results

Seventy patients undergoing CABG were enrolled in this study. Thirteen patients were excluded from the analysis, for a total of 57 patients. The reasons for exclusion were initial PCWP ≥ 18 mmHg (*n* = 5), initial CVP ≥ 15 mmHg (*n* = 1), arrhythmia (*n* = 1), and scheduling conflict (*n* = 6). There were 50 men and 7 women included in the study. There was no difference in the demographic variables between the responders and non-responders (Table 1). There were 21 responders (36.8%) and 36 non-responders (63.2%). There were 48 patients (84%) without regional wall motion abnormalities. Among the 9 patients with regional motion abnormalities, only 2 patients (one in the responders and one in the non-responders group) had regional wall motion abnormalities in the lateral basal wall. There were no patients with moderate to severe mitral annular calcification. Among the 56 patients with available diastolic function evaluation (simplified algorithm [14]), 10 had normal diastolic function (3 responders and 7 non-responders), 14 had mild diastolic dysfunction (4 responders and 10 non-responders), 25 had moderate diastolic dysfunction (12 responders and 13 non-responders), and 7 patients had severe diastolic dysfunction (1 responders and 6 non-responders), without difference in the number of responders between each grade (*P* = 0.3212).

**Table 1:** Characteristics of responder and non-responder patients after fluid infusion and leg raising

	Responders (*n* = 21)	Non-responders (*n* = 36)	*P* value
Age (years)	63.2 ± 8.9	66.0 ± 8.7	0.2534
Sex (men/women)	17/4	33/3	0.2345
Height (cm)	169.1 ± 8.7	168.4 ± 7.5	0.7374
Weight (kg)	80.9 ± 17.0	82.6 ± 16.2	0.7164
LVEF (%)	54.1 ± 10.4	53.3 ± 11.4	0.7954
LVH (*n*)	1 (4.8%)	2 (5.6%)	0.8913
Euroscore	1.6 (0.7, 3.0)	2.6 (1.1, 4.0)	0.1397
Renal insufficiency (*n*)	5 (23.8%)	10 (27.8%)	0.7428
HTA (*n*)	17 (81.0%)	32 (88.9%)	0.4053
Diabetes (*n*)	5 (23.8%)	13 (19.7%)	0.3351
Peripheral vascular disease (*n*)	1 (4.8%)	7 (19.4%)	0.1237
Previous stroke (*n*)	2 (9.5%)	5 (13.9%)	0.6281
COPD (*n*)	1 (4.8%)	8 (22.2%)	0.0812
Beta-blockers (*n*)	15 (71.4%)	28 (77.8%)	0.5911
Calcium channel blockers (*n*)	7 (33.3%)	16 (44.4%)	0.4095
ACE inhibitors (*n*)	10 (47.6%)	22 (61.1%)	0.3221
Diuretics (*n*)	5 (23.8%)	8 (22.2%)	0.8904
Statins (*n*)	18 (85.7%)	35 (97.2%)	0.1009

*ACE* angiotensin-converting enzyme inhibitors, *COPD* chronic obstructive pulmonary disease, *HTA* hypertension, *LVEF* left ventricular ejection fraction, *LVH* left ventricular hypertrophy

Baseline hemodynamic data, two-dimensional, pulsed wave, and tissue Doppler echocardiographic data after fluid bolus are shown for the responders and non-responders (Table 2). No difference in baseline hemodynamic parameters were found between responders and non-responders, except for SVV measured by Flotrac (13.95 ± 6.00% vs. 10.00 ± 4.35% for responders and non-responders respectively, *P* = 0.0076). No difference were found between responders and non-responders in baseline two-dimensional echocardiographic measurements of the right and left ventricle, except for left ventricular end-diastolic area (LVEDA) (13.58 ± 4.73 cm^2^ vs. 16.89 ± 5.38 cm^2^ for responders and non-responders respectively, *P* = 0.0335). No difference in baseline pulsed wave Doppler echocardiographic measurements of any components of the mitral, tricuspid, and pulmonary and hepatic venous flows were found between responders and non-responders. There was no difference in MV tissue Doppler measurements between responders and non-responders, including E/e′ ratio (8.68 ± 4.07 vs. 8.51 ± 2.83 in responders vs. non-responders, *P* = 0.8484).

**Table 2:** Hemodynamic, two-dimensional, pulsed wave, and tissue Doppler echocardiographic data for the responders and non-responders (baseline)

Variables	Responders (*n* = 21)	Non-responders (*n* = 36)	*P* value
Hemodynamic			
PCWP (mmHg)	11.30 ± 3.85	11.39 ± 3.45	0.9297
CVP (mmHg)	6.81 ± 3.66	7.58 ± 3.66	0.4444
PPV (%)	11.90 ± 7.91	8.53 ± 4.59	0.0921
SVV (%)	13.95 ± 6.00	10.00 ± 4.35	0.0076
PCWP ratio a/v	1.02 ± 0.14	1.04 ± 0.10	0.5729
RV ED pressure (mmHg)	10.85 ± 3.73	10.66 ± 3.74	0.8547
RV diastolic Dp/Dt (mmHg/s)	6.96 ± 4.07	8.00 ± 4.25	0.5458
Echo 2-D variables			
LV EDA (cm^2^)	13.58 ± 4.73	16.89 ± 5.38	0.0335
LV ESA (cm^2^)	6.99 ± 3.27	9.17 ± 3.99	0.0529
LV EF (%)	49.39 ± 9.70	45.98 ± 11.92	0.2999
RV EDA (cm^2^)	16.49 ± 3.32	17.65 ± 5.29	0.3306
RV ESA (cm^2^)	10.01 ± 2.96	11.37 ± 3.96	0.1828
RV FAC (%)	39.5 ± 11.39	37.05 ± 12.42	0.4697
MV Doppler			
E wave (cm/s)	0.57 ± 0.17	0.61 ± 0.13	0.2646
A wave (cm/s)	0.49 ± 0.14	0.50 ± 0.13	0.5928
E/A ratio	1.23 ± 0.43	1.26 ± 0.39	0.7302
PVF Doppler			
S wave (cm/s)	0.21 ± 0.08	0.22 ± 0.08	0.8391
D wave (cm/s)	0.31 ± 0.10	0.32 ± 0.09	0.7772
A wave (cm/s)	0.18 ± 0.07	0.16 ± 0.06	0.2237
S/D ratio	1.45 ± 0.36	1.48 ± 0.29	0.7431
TV Doppler			
E wave (cm/s)	0.34 ± 0.09	0.34 ± 0.08	0.9945
A wave (cm/s)	0.27 ± 0.08	0.28 ± 0.08	0.6440
E/A wave ratio	1.32 ± 0.35	1.28 ± 0.40	0.8168
HVF Doppler			
S wave (cm/s)	0.21 ± 0.08	0.22 ± 0.08	0.8391
D wave (cm/s)	0.126 ± 0.044	0.128 ± 0.050	0.8844
A wave (cm/s)	0.13 ± 0.07	0.12 ± 0.06	0.6159
S/D wave ratio	1.70 ± 0.55	1.76 ± 0.48	0.7073
MV tissue Doppler			
e′ wave (cm/s)	6.9 ± 2.3	7.7 ± 2.1	0.1669
E/e′ ratio	8.68 ± 4.07	8.51 ± 2.83	0.8484
TAPSE (mm)	2.20 ± 0.72	2.23 ± 0.57	0.8766

*CI* cardiac index, *CO* cardiac output, *ED* end-diastolic, *EDA* end-diastolic area, *EF* ejection fraction measured with the Simpson’s method, *ESA* end-systolic area, *FAC* fractional area change, *HVF* hepatic venous flow, *LV* left ventricular, *MV* mitral valve, *PCWP* pulmonary capillary wedge pressure, *PPV* pulse pressure variations, *PVF* pulmonary venous flow, *RV* right ventricular, *SPV* systolic pressure variations, *SVV* stroke volume variation measured by Flotrac, *TAPSE* tricuspid annular plane systolic excursion, *TV* tricuspid valve

Univariate logistic regressions models were used to assess for the influence of baseline hemodynamic parameters, two-dimensional, pulsed wave, and tissue Doppler echocardiographic measurements to predict fluid responsiveness. Only parameters with a *P* value < 0.20 in univariate analysis are shown in Table 3. Using this approach, we found that SVV variation measured by Flotrac and LVEDA were associated with fluid responsiveness. However, multivariate analysis revealed that SVV measured by Flotrac was the only independent variable to predict an increase in cardiac index more than 15% (OR = 1.196, 95% CI (1.028-1.393), *P* = 0.0208).

**Table 3:** Hemodynamic, two-dimensional, pulsed wave, and tissue Doppler echocardiographic parameters associated with fluids responsiveness

Variables	Univariate analysis, *B* (95% CI)	*P* value	Multivariate analysis, *B* (95% CI)	*P* value
Hemodynamic				
PPV (%)	1.099 (0.996-1.214)	0.0610		
SVV (%)	1.170 (1.032-1.327)	0.0143	1.196 (1.028-1.393)	0.0208
Echo 2-D variables				
LV EDA (cm^2^)	0.876 (0.771-0.995)	0.0410		
LV ESA (cm^2^)	0.831 (0.682-1.012)	0.0654		
RV ESA (cm^2^)	0.894 (0.758-1.055)	0.1838		

Only parameters with a *P* value < 0.20 in univariate analysis are presented. Among these parameters, SVV was the only parameter which remains in the final model (multivariate logistic regression analysis, with a *P* value < 0.05)

*EDA* end-diastolic area, *ESA* end-systolic area, *LV* left ventricular, *MV* mitral valve, *PPV* pulse pressure variations, *RV* right ventricular, *SPV* systolic pressure variations, *SVV* ejection volume variation measured by Flotrac

The performance of baseline hemodynamic and echocardiographic data to predict fluid responsiveness was evaluated by constructing ROC curves (Table 4). Area under the ROC curve was 0.70 for SVV measured by Flotrac using a threshold of 12% to predict fluid responsiveness (95% CI 0.55-0.85) and had a sensitivity of 66.7% with a specificity of 62.5%. However, there was a gray zone for SVV measured by Flotrac between 6 and 16%, which included 43% of the patients.

**Table 4:** Areas under the receiver operating characteristic (ROC) curves generated for hemodynamic and echocardiographic parameters to predict changes in cardiac index

Variables	Area under the ROC curve	95% confidence interval	Threshold	Sensitivity (%)	Specificity (%)	*P* value
Hemodynamic						
SVV (%)	0.6994	0.5486-0.8502	12	66.7	62.5	0.0096
PPV (%)	0.6343	0.4648-0.8038				0.1205
PCWP ratio a/v	0.5307	0.3603-0.7011				0.7240
Echocardiographic						
EDA (cm^2^)	0.6917	0.5290-0.8544	14.3	75.0	70.3	0.0209
MV E/e′ ratio	0.5231	0.3628-0.6835				0.7772

*EDA* (*cm*^*2*^) end-diastolic area, *MV E/e′ ratio* E/e*′* ratio of the mitral valve, *PCWP ratio a/v* ratio of the a/v wave of the pulmonary capillary wedge pressure tracing, *PPV* (*%*) pulse pressure variation, *SVV* (*%*) stroke volume variation measured with Flotrac

The change in right ventricular (RV) echocardiographic parameters, change in RV diastolic pressure over time (dP/dT), and RV end-diastolic pressure, from baseline to post fluid infusion are shown in Table 5. A statistically significant interaction term time × group for the LA diameter reveals a significant increase for the responder group but not for the non-responder group. For all other parameters, the interaction term was not significant. After fluid challenge, there was an overall (all groups) significant increase (all *P* < 0.05) in right atrial diameter, right ventricular end diastolic area, and left ventricular end diastolic area, without changes in right or left end systolic area and in right or left ventricular ejection fraction. There was also an increase in RV dP/dT and RV end diastolic pressure (all groups). A non-statistically significant interaction term (*P* = 0.7544) for the left ventricular E/e′ ratio of the mitral valve annulus was observed after fluid challenge, meaning that the two groups have the same evolution of this parameter in time (8.6 ± 4.07 to 9.44 ± 3.30 vs. 8.51 ± 2.83 to 9.03 ± 3.15 for responders and non-responders respectively, non-significant increase (*P* = 0.0835)).

**Table 5:** Echocardiographic parameters, RV dp/dt, and end diastolic RV pressure before and after fluid infusion

Variables	Group, responder (*n* = 21), non-responder (*n* = 36)	Baseline	After fluid infusion	*P* value (time × group interaction)	*P* value (time*)
LA diameter (mm)	Responder	4.14 ± 0.63	4.54 ± 0.67	*P* = 0.0166	*P* = 0.00023
	Non-responder	4.26 ± 0.67	4.35 ± 0.63		*P* = 0.2375
RA diameter (mm)	Responder	4.60 ± 0.79	4.92 ± 0.74	*P* = 0.5612	*P* = 0.0005
	Non-responder	4.71 ± 0.78	4.93 ± 0.83		
RV EDA (cm^2^)	Responder	16.49 ± 3.32	18.38 ± 4.37	*P* = 0.3100	*P* = 0.0037
	Non-responder	17.65 ± 5.29	18.56 ± 5.16		
RV ESA (cm^2^)	Responder	10.01 ± 2.96	10.69 ± 3.09	*P* = 0.9427	*P* = 0.0933
	Non-responder	11.37 ± 3.96	11.63 ± 3.80		
RV EF (%)	Responder	39.51 ± 11.39	41.74 ± 10.32	*P* = 0.3993	*P* = 0.1935
	Non-responder	37.05 ± 12.42	37.66 ± 9.79		
LV EDA (cm^2^)	Responder	13.58 ± 4.73	16.34 ± 4.33	*P* = 0.1498	*P* = 0.0015
	Non-responder	16.89 ±5.38	17.80 ± 4.67		
LV ESA (cm^2^)	Responder	6.99 ± 3.27	7.81 ± 3.04	*P* = 0.7628	*P* = 0.1808
	Non-responder	9.17 ± 3.99	9.50 ± 3.50		
LV EF (%)	Responder	54.83 ± 9.30	62.15 ± 8.60	*P* = 0.9211	*P* = 0.7039
	Non-responder	49.43 ± 12.78	49.46 ± 17.09		
MV E/e′ ratio	Responder	8.68 ± 4.07	9.44 ± 3.30	*P* = 0.7544	*P* = 0.0835
	Non-responder	8.51 ± 2.83	9.03 ± 3.15		
RV Dp/Dt	Responder	6.96 ± 4.07	10.14 ± 3.74	*P* = 0.8526	*P* = 0.006523
	Non-responder	8.00 ± 4.25	11.17 ± 5.02		
RV ED pressure (mmHg)	Responder	10.85 ± 3.73	14.90 ± 3.78	*P* = 0.9107	*P* < 0.0001
	Non-responder	10.66 ± 3.74	14.75 ± 3.93		

*ED pressure* end-diastolic pressure, *EDA* end-diastolic, *ESA* end-systolic, *LA* left atrium, *LV* left ventricle, *MV* mitral valve, *RA* right atrium, *RV dP/dt* change in right ventricular pressure over time, *RV* right ventricle

^*^Overall effect time *P* value except for LA diameter because of the statistically significant interaction time × group

## Discussion

In this pilot study, we observed that predictor of fluid responsiveness was unrelated to left ventricular diastolic function parameters. Although left ventricular diastolic function may play a role in fluid responsiveness, our observations do not support the hypothesis that the evaluation of left ventricular diastolic function parameters is relevant in the prediction of fluid responsiveness. Indeed, there was neither difference in the E/e′ ratio of the mitral valve between responders and non-responders nor there was any difference in Doppler measurements of mitral valve inflow, pulmonary venous flow (PVF), and tissue Doppler components of MV annulus velocities. We also observed a lack of difference for Doppler measurements of tricuspid valve inflow, and hepatic venous flow (HVF) between responders and non-responders. The lack of difference in RV dP/dT of the diastolic filling and RV end-diastolic pressure between responders and non-responders are not in favor for a predominant role of RV diastolic function to predict preload responsiveness. Taken together, assessment of multiple parameters of biventricular diastolic function, including the E/e′ ratio of the mitral valve, does not increase our ability to predict fluid responsiveness in this population of patients undergoing CABG.

As confirmed by other investigators, the absolute values of CVP and PCWP (static parameters) were not useful in predicting FR in these patients [15]. In contrast to another study [2], we found that LV end-diastolic area (static parameter) had a significant correlation with fluid responsiveness by univariate analysis. Only SVV measured by Flotrac was independently correlated with fluid responsiveness, as assessed with multivariate analysis. However, SVV had only a modest sensitivity and specificity to predict FR, and almost half of the patients are in a gray zone, which limits its usefulness as a predictor of FR.

There are very few studies evaluating the role of diastolic function to predict fluid responsiveness [9, 10]. In a previous study, Lattik et al. [10] observed that a mitral E/A ratio < 1.26 was superior to any hemodynamic variables in predicting fluid responsiveness in CABG patients (area under the ROC curve of 71%). They suggested that measurements of E/A ratio may be useful in determining the position of an individual patient on the LV pressure/volume curve. When that study was performed, TDI was not measured because unavailable on TEE equipment in 2002. In a subsequent study, Roy et al. [9] found that the a/v wave ratio of the PCWP tracing > 1 was associated with preload responsiveness (area under the ROC curve of 0.89%), which, according to the authors, is a reflection of a normal diastolic function. The difference in these studies and the present one could be explained by differences in population studied, the type and volume of fluid infused, the rate of fluid administration, and time of measurements. However, in neither of these previous studies, assessment of multiple parameters of biventricular diastolic function to predict preload responsiveness has been performed. Even if the hypothesis that a compliant heart can accommodate further volume and thus increase cardiac output is appealing, our analysis of multiple parameters of LV and RV diastolic function could not support these prior results [9, 10].

The hemodynamic response to a fluid loading is related to the Frank-Starling function curve. However, it depends also on the venous return to the heart, which determines if the administered volume is actually getting to the heart during diastole to increase preload [16]. Venous return is a function of stressed vascular volume, venous compliance and resistance, and the pressure gradient between the periphery mean systemic venous pressure (MSVP) and the right atrial pressure (RAP) [16]. The peripheral venous system has a large vascular capacitance and a linear compliance, whereas the diastolic pressure of the heart is curvilinear [16]. These differences in compliance could affect the venous return pressure gradient, because RAP could rise faster than MSVP [17], causing the net pressure gradient for venous return to decrease [17]. We did not measure the mean systemic venous pressure in our study, and consequently, we cannot determine if the MSVP-RAP gradient was different between responders and non-responders. Because preload responsiveness depends on many factors including the systolic and diastolic function, the position on the Frank Starling curve and venous return, for both of the right and left ventricle, and also on a decrease in afterload induced by hemodilution [18], it is difficult to assess the effect of RV and LV diastolic function in isolation on the hemodynamic response to fluid challenge. The effect of diastolic function may be less important than the combination of all the factors mentioned above.

This study has several limitations. First, no prestudy power analysis was performed in this exploratory study. Pilot study like this one serves to identify opportunities to test hypotheses with prospective registration trial with adequate sample size. Second, the results apply to patients undergoing CABG with a closed chest, under general anesthesia and mechanical ventilation, in normal sinus rhythm, preserved right ventricular function, and cannot be generalized to other situations. Third, although more recent guidelines have been published to assess diastolic function [19], it may be difficult to use because some of the parameters, such as left atrial volume, and peak tricuspid regurgitation measurements, are not validated with TEE. Instead, we evaluated TDI parameters of the mitral annulus as well as Doppler tricuspid and mitral flow parameters and used a simplified algorithm to grade the diastolic function [14]. The E/e′ ratio has been shown to correlate as an index of filling pressures and is a preload independent index of LV relaxation. As the E/e′ ratio increases, the severity of diastolic dysfunction becomes more pronounced [19]. The lack of differences in all these parameters between responders and non-responders suggests a minor role of right and left ventricular diastolic function to predict fluid responsiveness in our population. Moreover, there was no difference in the number of responders and non-responders between the different grades of diastolic dysfunction. Fourth, we used crystalloids instead of colloids, used in many of the preload responsiveness studies, which may explain the lower rate of fluid responsiveness obtained in our study. However, the fluid infusion was done within a short period (10 min), and measures were taken within 5 min after fluid infusion, which should limit the difference between colloids and crystalloids to increase preload [20]. Moreover, a meta-analysis by Toscani et al. [20] suggests that there is no difference in the proportion of responders when the time of measurements was assessed at the end of the fluid challenge or between 1 and 10 min after the fluid challenge. Our time of measurement was 5 min after the end of the fluid challenge and was consistent for all the patients.

In addition, we made the measurements with the legs raised, which adds around 150 mL of volume [21], to the fluid challenge. In this manner, we wanted to ensure that the volemic load was sufficient to increase venous return and cardiac output. Even if there is a possibility that this amount of fluid challenge may have produced right ventricular overload which may limit the fluid responsiveness [22] in our study (only a third were responders), we did not find differences between the two groups for right ventricular dimension, right ventricular ejection fraction, right ventricular diastolic pressure waveform analysis (dP/dT), CVP, and end diastolic right ventricular pressure after fluid challenge. Then, the possibility of RV overload is unlikely to explain preload unresponsiveness in this study. The effect of fluid challenge on stroke volume/cardiac output remains difficult to explain within the parameters measured. The concept of fluid responsiveness in itself does not mean that every fluid responder needs further fluid infusion, considering the deleterious effect of fluid overload on organs’ perfusion [23–28].

In conclusion, in this pilot study, assessment of multiple parameters of left and right ventricular diastolic function, including E/e′ ratio of the mitral valve annulus measured by TEE, is not a predictor of fluid responsiveness undergoing CABG. The SVV measured by the Flotrac/Vigileo system was the most useful parameter to predict fluid responsiveness; however, almost half of the patients are in a gray zone for this parameter, which decreases its usefulness to guide fluid therapy. Further studies are needed to confirm the results of this exploratory study.

## Materials and methods

### Population

After approval by the Montreal Heart Institute Research Ethics and New Technology Development Committee (F1-4651) and with informed consent, 70 patients over 18 years old undergoing elective coronary artery bypass grafting (CABG) surgery between January 2016 and September 2017 were enrolled. No prestudy power analysis was performed. After that period, the department elected to terminate this exploratory study and analyze the data. The study was retrospectively registered at Clinical Trials.gov (provided by the US National Library of Medicine) on March 21, 2016, with the Identifier: NCT02714244. Patients with more than mild valvular heart disease, intracardiac shunts, preoperative arrhythmia (non-sinus rhythm), decompensated heart failure, or pulmonary hypertension (mean pulmonary arterial pressure > 25 mmHg), renal insufficiency defined as creatinine clearance of less than 30 mL/min or any contraindications for TEE (such as esophageal disease or unstable cervical spine) were excluded. Central venous pressure (CVP) and pulmonary capillary wedge pressure (PWCP) were assessed before fluid administration and patients with high values (CVP ≥ 15 mmHg or PWCP ≥ 18 mmHg) were excluded because of risk of fluid overload in these patients.

### Methods

Premedication, induction, and maintenance of anesthesia were achieved at the discretion of the attending anesthetist with the exception of the requirement for the use of isoflurane inspired concentration of 1%. Lungs were ventilated by intermittent positive-pressure ventilation with a fraction of inspired oxygen of 1.0 by use of an Ohmeda volume-cycled ventilator (Ohmeda, Helsinki, Finland). A respiratory rate of 8 breaths/min with an inspiratory/expiratory ratio of 1:2 and a tidal volume of 8 mL/kg were kept constant for the entire experiment. No positive end-expiratory pressure was applied. Usual monitoring was installed, including a 5-lead electrocardiogram, pulse oximeter, peripheral venous line, radial arterial line, 15-cm 3-lumen catheter (CS-12703, Arrow international Inc., Reading, CA), and fast-response thermodilution pulmonary artery catheter (Swan-Ganz catheter 7.5 Fr; Edwards Lifesciences Corporation, Irvine, CA). A FloTrac (Edwards Lifesciences Corporation, Irvine, CA) sensor was added to the arterial line and was analyzed by the Vigileo monitor (Edwards Lifesciences Corporation, Irvine, CA). A 5.0-MHz TEE omniplane probe (Vivid 7; GE Healtcare System, Milwaukee, WI) was inserted by the anesthesiologist after induction of general anesthesia.

### Hemodynamic parameters

After the induction of anesthesia, central line insertion and hemodynamic stability, the following parameters were recorded: arterial blood pressure, cardiac output (CO), right ventricular pressure, CVP, pulmonary artery pressure (PAP), PCWP, and respiratory-induced SVV. The CO was assessed with thermodilution technique using three injections of dextrose 5% (10 mL) at room temperature at end expiration. The average of three measurements with less than 10% difference between each was noted. Cardiac output response measured with the Swan-Ganz catheter was considered the gold standard to assess preload responsiveness. FloTrac was used to determine SVV. The PPV was calculated from the arterial line tracing on the monitor (Philips, Intellivue G5 M1019A).

### Echocardiographic parameters

We obtained a complete baseline TEE exam, including the evaluation of diastolic function of the right and left ventricle, according to the ASE/SCA Guidelines for performing a comprehensive intraoperative multiplane TEE examination [8, 29]. The lateral mitral annulus (e′) was measured using TDI, along with measurement of the E wave peak diastolic velocity with pulsed-wave Doppler, and the E/e′ was derived. We also obtained a TEE exam (four chamber, trans-gastric, mitral flow Doppler, pulmonary venous flow Doppler, hepatic venous flow Doppler, and tricuspid Doppler flow) before and after volume infusion. In addition, we determined diastolic dysfunction using a simplified algorithm previously described by Swaminathan et al. [14].

- Normal diastolic function: e′ ≥ 10 cm·s^−1^
- Grade 1 diastolic dysfunction (mild): e′ < 10 cm·s^−1^; E/e′ ≤ 8
- Grade 2 diastolic dysfunction (moderate): e′ < 10 cm·s^−1^; E/e′ = 9-12
- Grade 3 diastolic dysfunction (severe): e′ < 10 cm·s^−1^; E/e′ > 13

A single National Board of Echocardiography (NBE) board-certified anesthetist obtained the echocardiographic images. If any doubt arose about the interpretation of an echocardiographic measurement or hemodynamic waveform interpretation, the advice of a second anesthetist was obtained.

### Protocol

All parameters were measured before chest opening and during hemodynamic stability in a supine position. The first measurement (T1) was carried within the 15 min that preceded a rapid volume infusion of 500 mL of Lactate Ringer® over 10 min. The second measurement (T2) was obtained within a maximum of 5 min after the bolus. Because T2 coincided with the time of asepsis, patients’ legs were raised, causing further increase of venous return. The legs were raised for the duration of the measurements for each patient. Fluid infusion was stopped if the PCWP was ≥ 20 mmHg during the protocol and such patients were excluded from the study. Fluid responsiveness was defined as an increase in cardiac index of more than 15% [30].

### Statistics

Continuous variables are summarized using mean ± standard deviation, or median (Q1, Q3), according to the distribution of the variable, while categorical variables are described using frequencies and percentages. Demographic data were compared between responders and non-responders using Student *t* test or Mann-Whitney-Wilcoxon test as appropriate for continuous variables, and chi-square test for categorical variables. The number of responders and non-responders for each grade of diastolic dysfunction (normal to severe) was compared using chi-squared test. Comparison of groups for diastolic function parameters (including mitral E/e′ ratio), 2-D and Doppler echocardiographic measurements, and hemodynamic measurements was performed with a Students *t* test. Then, using univariate logistic regression models, we explored the potential predictors of preload responsiveness among the same echocardiographic, and hemodynamic values. Only parameters with a *P* value < 0.20 in univariate analysis were included in the stepwise selection procedure of a multivariate logistic regression model. Receiver operating characteristic (ROC) curves were also generated for hemodynamic and echocardiographic parameters. Because the ROC curve methodology does not take into account, the existence of overlap between positive and negative fluid challenges, we also use the gray zone approach. This methodology avoids the binary response proposed by the ROC curves and proposed a low cut-off value that excludes positive fluid challenge in 90% of patients, whereas a high cut-off value predicts positive fluid challenge in 90% of cases [31]. Two-way repeated measures ANOVA model was used to compare the evolution of the two groups in terms of right and left ventricular and atrial parameters, right ventricular pressure over time (dP/dT), right ventricular end-diastolic pressure, and E/e′ ratio of the mitral valve before and after fluid challenge. The interaction term was tested, and comparison pre vs. post fluid challenge was produced in each group in case of statistically significant interaction. A two-tailed *P* value < 0.05 was considered statistically significant. All statistical analysis was performed using the SAS version 9.4 software (SAS Institute, NC).

## Data Availability

The datasets used and/or analyzed during this current study are available from the corresponding author on reasonable request.
